# Supplementary material for: MetaRibo-Seq measures translation in microbiomes
Source: Nat Commun. 2020 Jun 29;11:3268. doi: 10.1038/s41467-020-17081-z (PMC7324362; doi:10.1038/s41467-020-17081-z)
Supplement: Supplementary file 10 — Supplementary Data 7 [file 41467_2020_17081_MOESM10_ESM.zip › File2/Confidence_High_Taxonomy/83290_out.krona.html]

Javascript must be enabled to view this page.

members
magnitude
magnitudeUnassigned
count
unassigned
taxon
rank

83290\_out

143

2
143
superkingdom

phylum
143
976

class
200643
143

143
171549
order

family
815
143

143
816
genus
43

SRS011239\_contig\_number\_14130SRS013940\_contig\_number\_contig-100\_2373.130988SRS014923\_contig\_number\_contig-100\_2566.226394SRS015782\_contig\_number\_2163SRS017433\_contig\_number\_2577SRS018313\_contig\_number\_contig-100\_3412.77976SRS019601\_contig\_number\_444SRS020869\_contig\_number\_12323SRS022137\_contig\_number\_15857SRS023346\_contig\_number\_1991SRS023715\_contig\_number\_23011SRS024132\_contig\_number\_49706SRS024388\_contig\_number\_contig-100\_3908.88038SRS043701\_contig\_number\_2704SRS049896\_contig\_number\_13192SRS050026\_contig\_number\_2658SRS051031\_contig\_number\_contig-100\_3085.202041SRS052027\_contig\_number\_11659SRS054352\_contig\_number\_23241SRS057480\_contig\_number\_contig-100\_2448.77291SRS058770\_contig\_number\_422SRS064276\_contig\_number\_10403SRS064557\_contig\_number\_2379SRS065176\_contig\_number\_640SRS077194\_contig\_number\_1221SRS077454\_contig\_number\_1270SRS077502\_contig\_number\_6789SRS077552\_contig\_number\_15496SRS078419\_contig\_number\_17298SRS097920\_contig\_number\_contig-100\_2287.191530SRS097958\_contig\_number\_18097SRS104311\_contig\_number\_963SRS1055022\_contig\_number\_21149SRS140513\_contig\_number\_9665SRS142980\_contig\_number\_2742SRS143148\_contig\_number\_8169SRS144297\_contig\_number\_contig-100\_4004.94413SRS147272\_contig\_number\_contig-100\_840.841SRS148817\_contig\_number\_2499SRS148874\_contig\_number\_4411SRS893292\_contig\_number\_contig-100\_5980.40087SRS893295\_contig\_number\_11795SRS893341\_contig\_number\_11902

1
1263044
species

SRS064757\_contig\_number\_contig-100\_1612.130063

species

SRS011405\_contig\_number\_8692SRS015694\_contig\_number\_4143SRS147425\_contig\_number\_1548
3
371601

626929
1

SRS019787\_contig\_number\_1801
species

817
1

SRS098881\_contig\_number\_21029
species

1262750
1

SRS1055076\_contig\_number\_1350
species


SRS063190\_contig\_number\_11054
species
1638780
1


SRS015960\_contig\_number\_748SRS143895\_contig\_number\_35676
species
46506
2

species

SRS065397\_contig\_number\_contig-100\_733.82460SRS147653\_contig\_number\_874
2
310297

3
338188
species

SRS045713\_contig\_number\_58SRS1041116\_contig\_number\_8861SRS893300\_contig\_number\_11489

species

SRS147377\_contig\_number\_10616
1
1841855


SRS019693\_contig\_number\_3687
species
820
1

species

SRS014855\_contig\_number\_12715SRS043411\_contig\_number\_12495SRS050801\_contig\_number\_2642SRS058268\_contig\_number\_114
4
2292956

2292955
1

SRS020622\_contig\_number\_448
species

1
671267
species

SRS050925\_contig\_number\_29145

species

SRS014736\_contig\_number\_7538SRS020508\_contig\_number\_332SRS022524\_contig\_number\_10205SRS045826\_contig\_number\_11673SRS046810\_contig\_number\_contig-100\_19.20SRS048870\_contig\_number\_15260SRS052576\_contig\_number\_348SRS054905\_contig\_number\_5337SRS054956\_contig\_number\_8656SRS056519\_contig\_number\_737SRS077231\_contig\_number\_13726SRS098717\_contig\_number\_739SRS101376\_contig\_number\_22731SRS1041138\_contig\_number\_contig-100\_3496.68224SRS1041141\_contig\_number\_contig-100\_3002.39641SRS104485\_contig\_number\_431SRS143523\_contig\_number\_3706SRS146813\_contig\_number\_25056SRS147022\_contig\_number\_691SRS147919\_contig\_number\_1399SRS148424\_contig\_number\_28413SRS893259\_contig\_number\_4816
22
1739517

32
28116
species

SRS013215\_contig\_number\_410SRS013476\_contig\_number\_19796SRS015578\_contig\_number\_26761SRS015579\_contig\_number\_8566SRS016840\_contig\_number\_1592SRS017191\_contig\_number\_5513SRS017622\_contig\_number\_391SRS018936\_contig\_number\_10440SRS019496\_contig\_number\_13739SRS024549\_contig\_number\_257SRS046712\_contig\_number\_337SRS048060\_contig\_number\_8607SRS050422\_contig\_number\_11910SRS053573\_contig\_number\_1117SRS057717\_contig\_number\_792SRS064232\_contig\_number\_1271SRS074964\_contig\_number\_914SRS078665\_contig\_number\_1075SRS104084\_contig\_number\_11155SRS104693\_contig\_number\_11558SRS1055034\_contig\_number\_10111SRS1055056\_contig\_number\_contig-100\_1210.78410SRS1055095\_contig\_number\_2131SRS140645\_contig\_number\_166SRS143417\_contig\_number\_14866SRS143466\_contig\_number\_31760SRS143722\_contig\_number\_16SRS145308\_contig\_number\_2512SRS149244\_contig\_number\_10004SRS893231\_contig\_number\_4186SRS893342\_contig\_number\_1739SRS893369\_contig\_number\_8733


SRS104327\_contig\_number\_198
species
1263054
1

821
1

SRS045645\_contig\_number\_20306
species

1907658
1

SRS1041112\_contig\_number\_661
species

species

SRS013951\_contig\_number\_32850SRS014412\_contig\_number\_19365SRS014613\_contig\_number\_18513SRS019030\_contig\_number\_14573SRS147039\_contig\_number\_1253SRS147614\_contig\_number\_2058SRS893383\_contig\_number\_31932
7
47678

species

SRS015854\_contig\_number\_3829SRS016095\_contig\_number\_13188SRS077127\_contig\_number\_1252SRS1041139\_contig\_number\_3299SRS143598\_contig\_number\_272SRS148784\_contig\_number\_1123SRS893279\_contig\_number\_4540SRS893288\_contig\_number\_1684
8
818


SRS055982\_contig\_number\_310SRS058070\_contig\_number\_12024SRS148721\_contig\_number\_11828
species
1796613
3

species

SRS046717\_contig\_number\_1725
1
291645


SRS053649\_contig\_number\_1780
species
329854
1
